# Supplementary material for: NFIC regulates ribosomal biology and ER stress in pancreatic acinar cells and restrains PDAC initiation
Source: Nat Commun. 2023 Jun 23;14:3761. doi: 10.1038/s41467-023-39291-x (PMC10290102; doi:10.1038/s41467-023-39291-x)

I. Cobo et al. NFIC regulates ribosomal biology and ER stress in pancreatic acinar cells and restrains PDAC initiation

SUPPL. MATERIAL

SUPPL. FIGURE

A

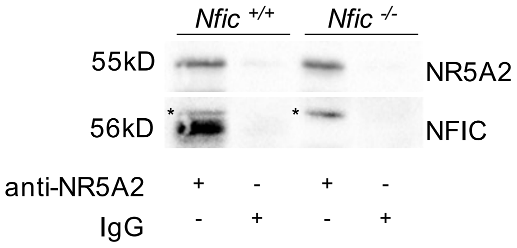

B

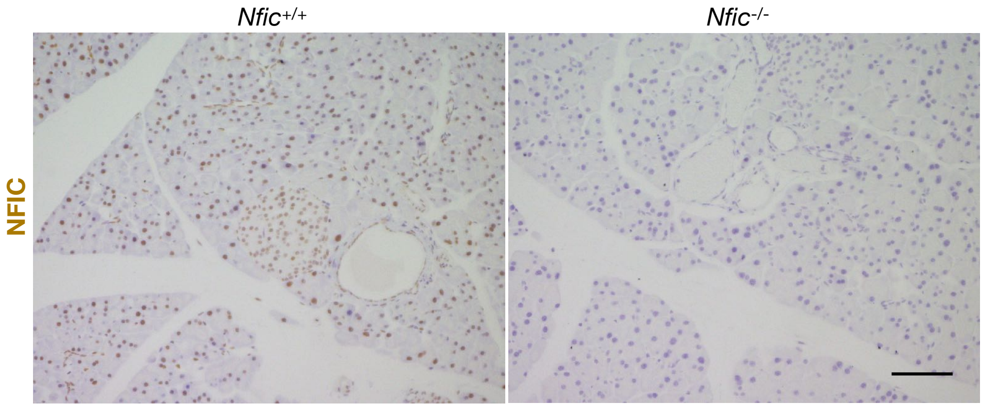

Supplementary Figure 1. NFIC validation experiments. (A) Validation of the specificity of the interaction of NR5A2 and NFIC in the pancreas of adult mice. Immunoprecipitation-western blotting analysis showing that the two proteins are part of the same complex in *Nfic* WT mice. In contrast, NR5A2 immunoprecipitates of *Nfic*$^{-/-}$ pancreata do not contain proteins recognized by anti-NFIC antibodies (representative image of 2 independent experiments). * indicates a non-specific band. (B) Immunohistochemical validation of the antibody recognizing NFIC used in the study. NFIC is detected in epithelial cells in the pancreas of wild-type (WT) mice but not in *Nfic*$^{-/-}$ mice (representative image of 3 independent experiments).

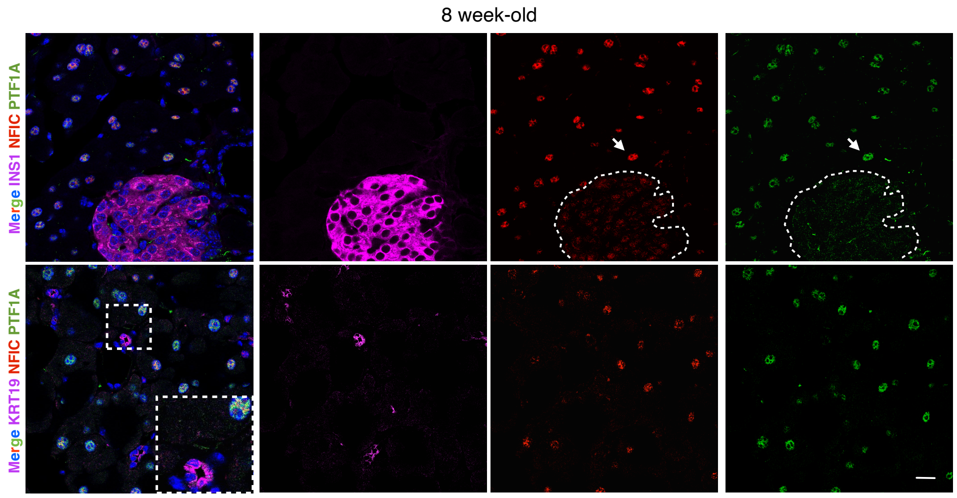

Supplementary Figure 2. In the adult mouse pancreas, NFIC is expressed at highest levels in acinar cells and at lower levels in ductal and endocrine cells. Expression analysis of NFICin the pancreas of 8 week-old wild-type mice using triple IF on 4% PFA-fixed sections. NFIC is expressed at higher levels in acinar cells than in endocrine cells (INS1^{+}) and it is undetectable inductal cells (KRT19^{+}). Square with dotted lines denotes the magnified area. Arrow denotes acinarcell. One representative image of 4 wild-type pancreata is shown

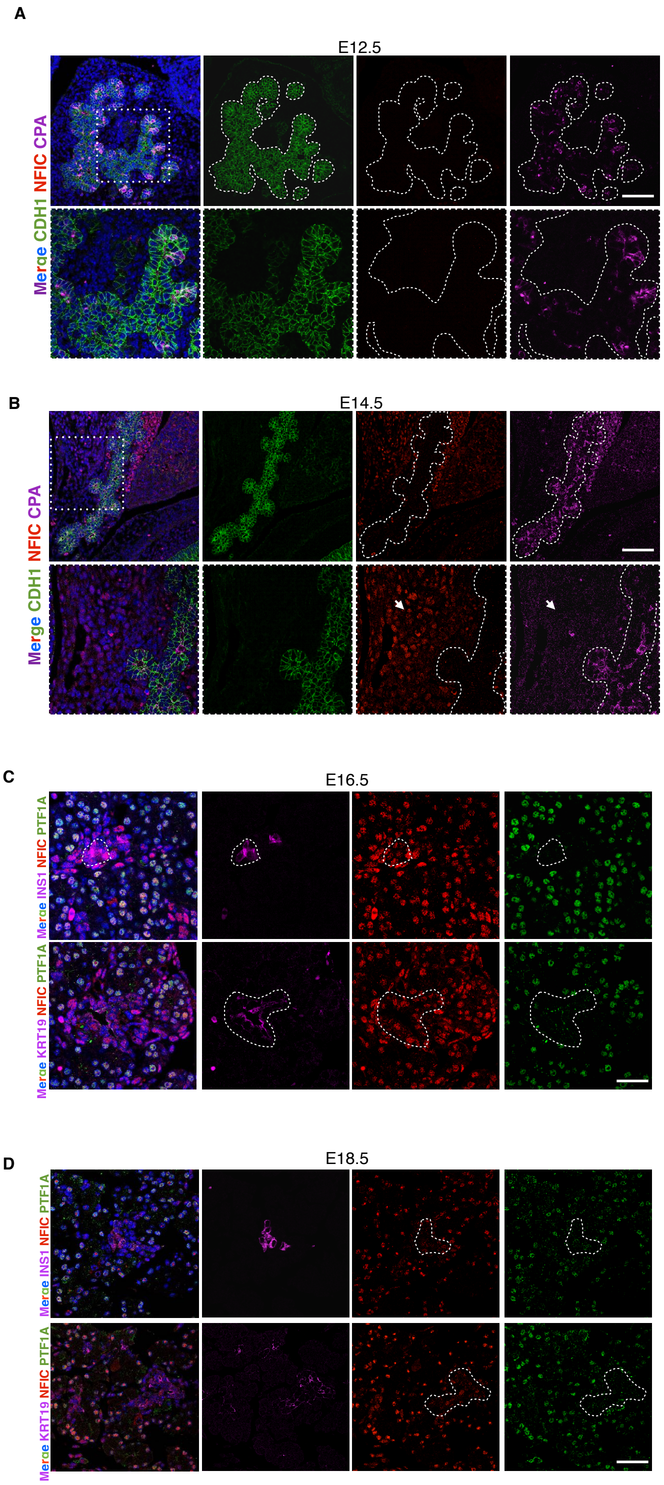

Supplementary Figure 3. NFIC is expressed at late stages of pancreatic development. (A,B)Expression analysis of NFIC in the pancreas of E12.5 (A) and E14.5 (B) wild-type embryos usingtriple IF on 4% PFA-fixed sections shows undetectable levels of NFIC. Expression of CDH1 andCPA was used to trace pancreatic progenitors. Broken lines delineate epithelial cells of the embryonic pancreas; arrows point to cells outside the embryonic pancreas showing the expression of NFIC in non-pancreatic cells in E14.5 embryos. (C,D) Expression of NFIC in the pancreas of E16.5 (C) and E18.5 (D) wild-type embryos using triple IF with antibodies detecting PTF1A, INS1, and KRT19. The expression of NFIC in acinar (PTF1A^{+}), endocrine (INS1^{+}) and ductal cells (KRT19^{+}) is shown. One representative image of 3 wild-type pancreata is shown. Square with broken lines denotes the region magnified.

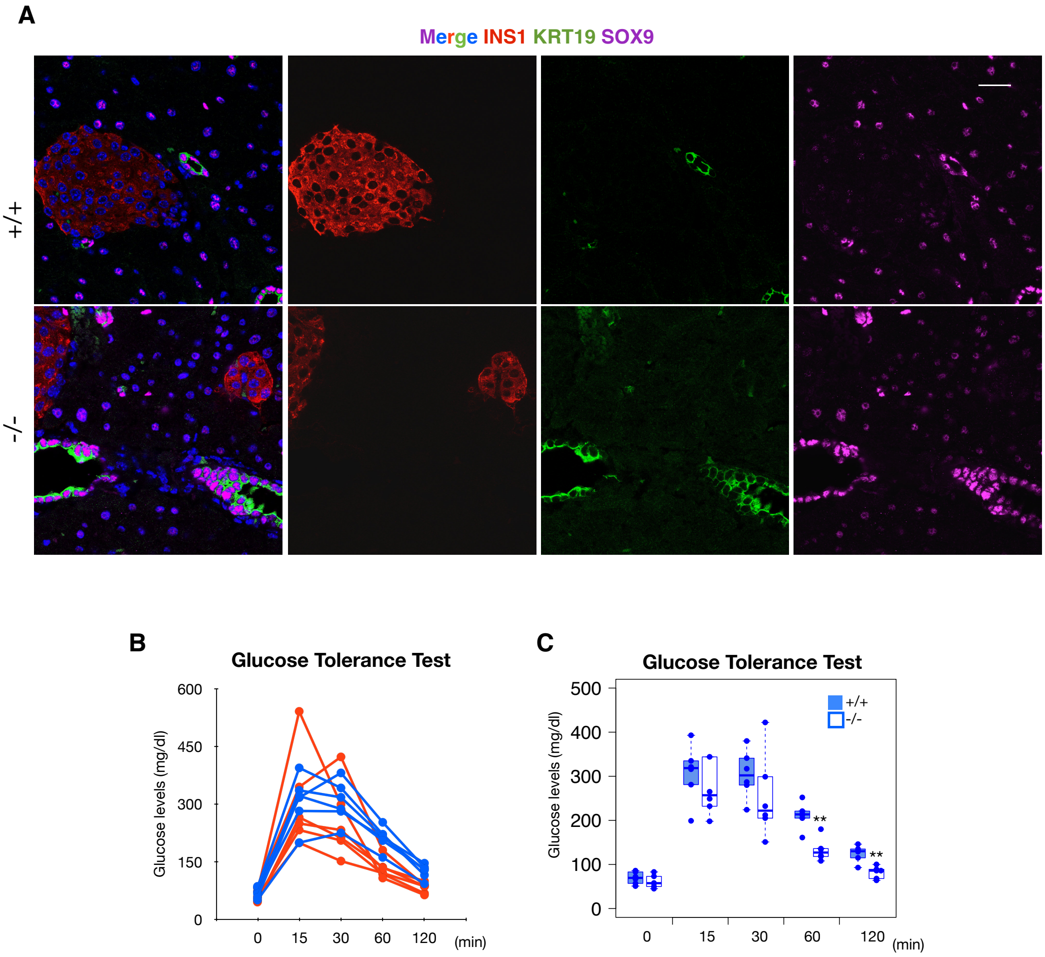

Supplementary Figure 4. *Nfic*$^{-/-}$ mice display no major abnormalities in ductal or endocrinecells and have a normal response to glucose overload. (A) IF analysis of INS1, KRT19 and SOX9 expression in wild-type and *Nfic*$^{-/-}$ pancreata (8-10 weeks). A representative image of 3 independent experiments is shown. (B,C) Glucose tolerance test in wild-type and *Nfic*$^{-/-}$ mice (11-17 weeks) showing similar glucose levels in mice of both genotypes at 0, 15 and 30 minutes but reduced glucose levels in *Nfic*$^{-/-}$ mice at 60 and 120 min (n=6 male mice/genotype). Fasting glucose levels were measuredbefore and after intra-peritoneal injection of glucose (2g/kg of body mass). Data for each individualmice (B) and grouped by genotype (C) are shown. In (C), two-sided Mann-Whitney U test was used to calculate statistical significance. P<0.01(**). At 0 min, P=0.37; at 15 min, P=0.58; at 30 min, P=0.24; at 60 min, P=0.004; at 120 min, P= 0.008. Boxplots are displayed showing the minimum, the maximum, the sample median, and the first and third quartiles. Source data are provided as a source data file.

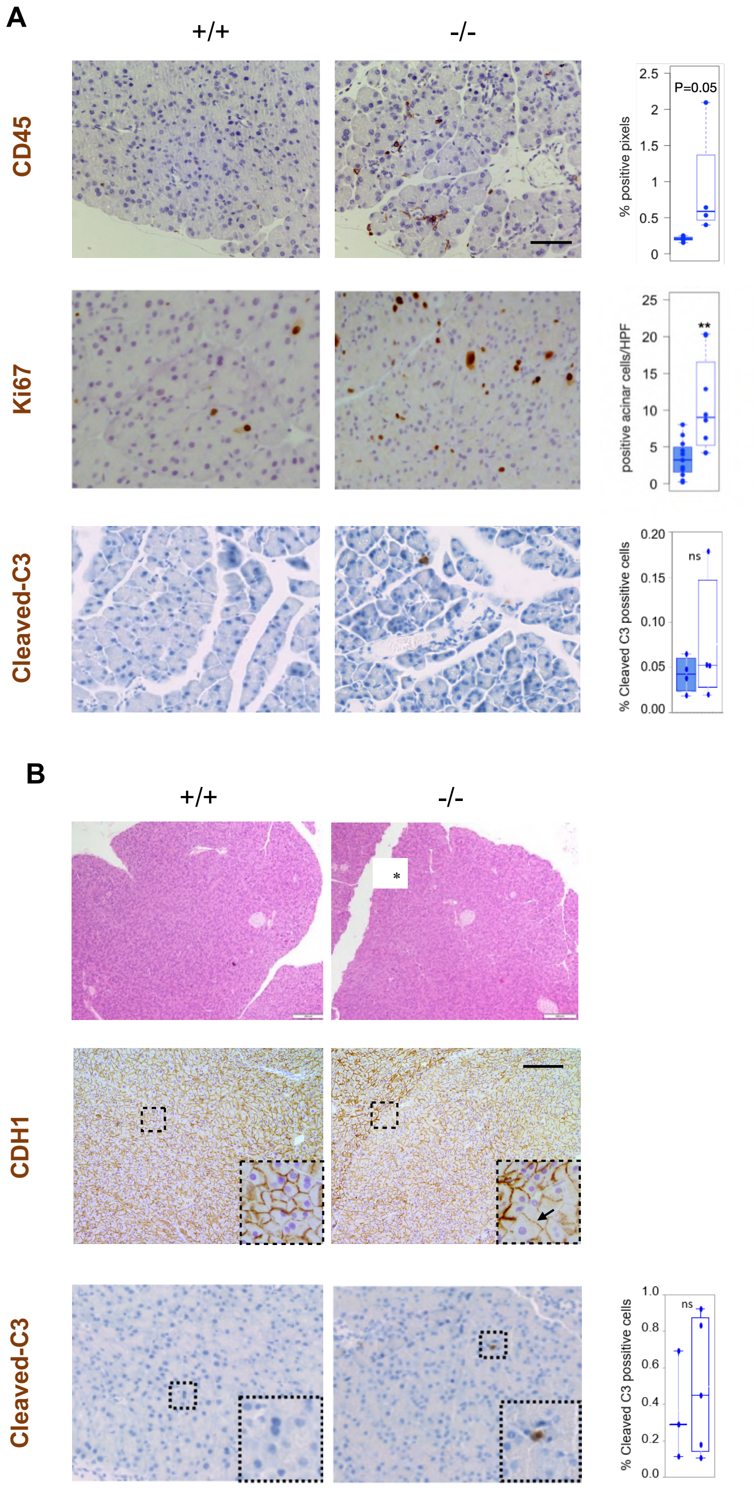

Supplementary Figure 5. Histological and immunohistochemical analysis of the pancreas of young adult (8-10 week-old) (A) and older (20-25 week-old) (B) WT and *Nfic*$^{-/-}$ mice. (A) IHC and quantitative analysis of leukocyte infiltration ($n \geq 3$ mice/genotype), acinar cell proliferation (Ki67) ($n \geq 8$ mice/genotype), and apoptosis (cleaved caspase 3, cC3) (n=4 mice/genotype) in the pancreas of wild-type and *Nfic*$^{-/-}$ mice (CD45, P=0.05; KI67 P=0.004; cC3, P=0.69). (B) H-E staining of old WT and *Nfic*$^{-/-}$ pancreata and IHC analysis of CDH1 expression and cC3 ($n \geq 3$ mice/genotype). For IHC analysis, random images (n=10) were taken from each pancreas and only unequivocally positive acinar cells were considered. Two-sided Mann-Whitney U test was used to calculate statistical significance. Boxplots show the minimum, the maximum, the sample median, and the first and third quartiles. Source data are provided as a source data file.

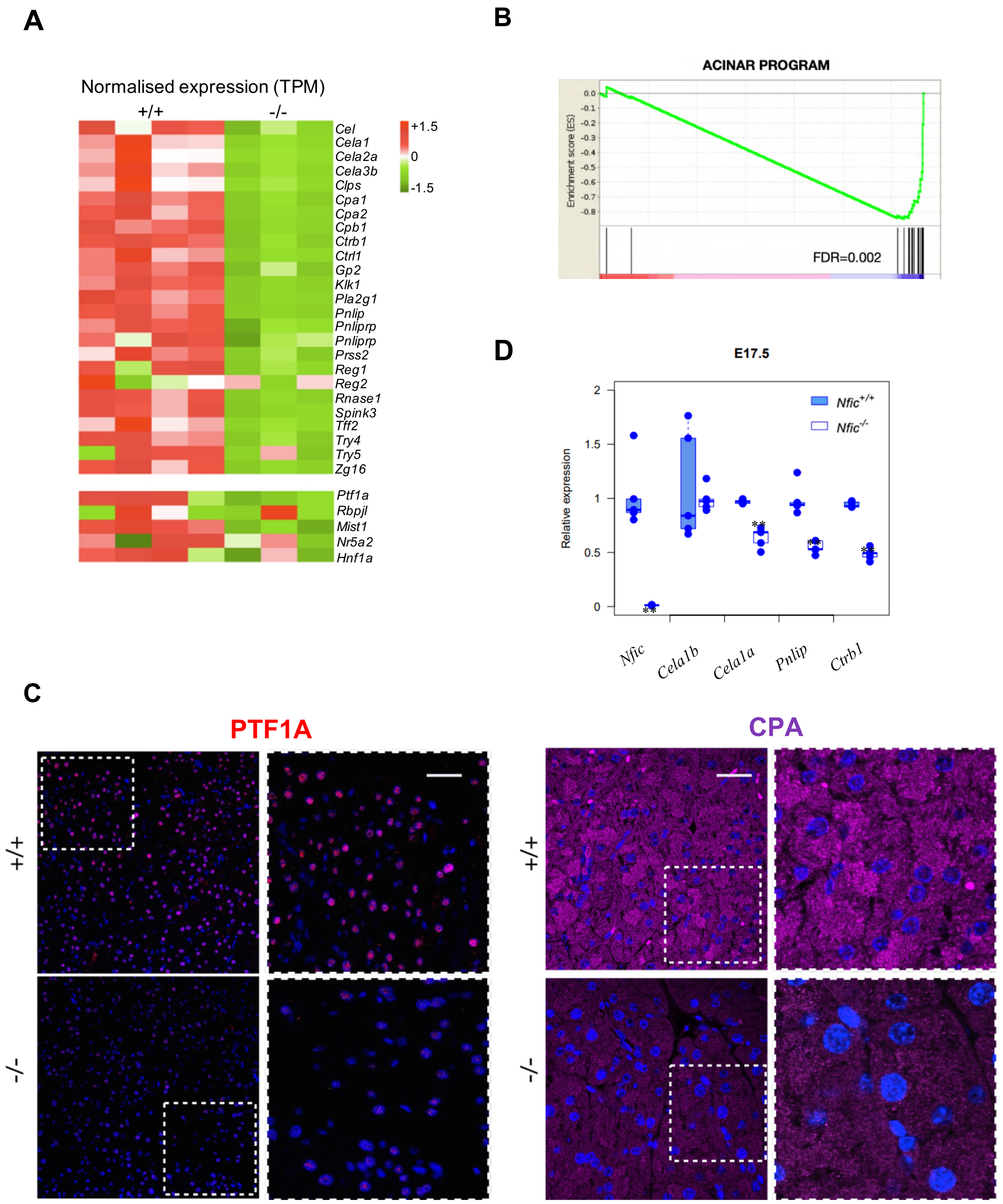

Supplementary Figure 6. NFIC is required for normal acinar cell differentiation. (A) Heatmap showing the expression of transcripts coding for digestive enzymes, secretory granule components, and acinar transcription factors in WT and *Nfic*$^{-/-}$ pancreata (RNA-Seq data). (B) GSEA enrichment plot showing the down-regulation of the acinar signature defined by Masui *et al*. in *Nfic*$^{-/-}$ pancreata. (C) IF analysis of PTF1A and CPA expression in WT and *Nfic*$^{-/-}$ pancreata showing regions of the exocrine parenchyma with reduced expression of both proteins. One representative image is shown (n=3 mice/genotype). (D) RT-qPCR analysis of expression of digestive enzyme transcripts in the pancreas of E17.5 WT and *Nfic*$^{-/-}$ pancreata (n=5 mice/genotype; *Nfic,* P=0.007; *Cela2a,* P=0.008; *Pnlip,* P=0.007; *Ctrb1,* P=0.008; two-sided Mann-Whitney U test). Boxplots are displayed showing the minimum, the maximum, the sample median, and the first and third quartiles. Source data are provided as a source data file.

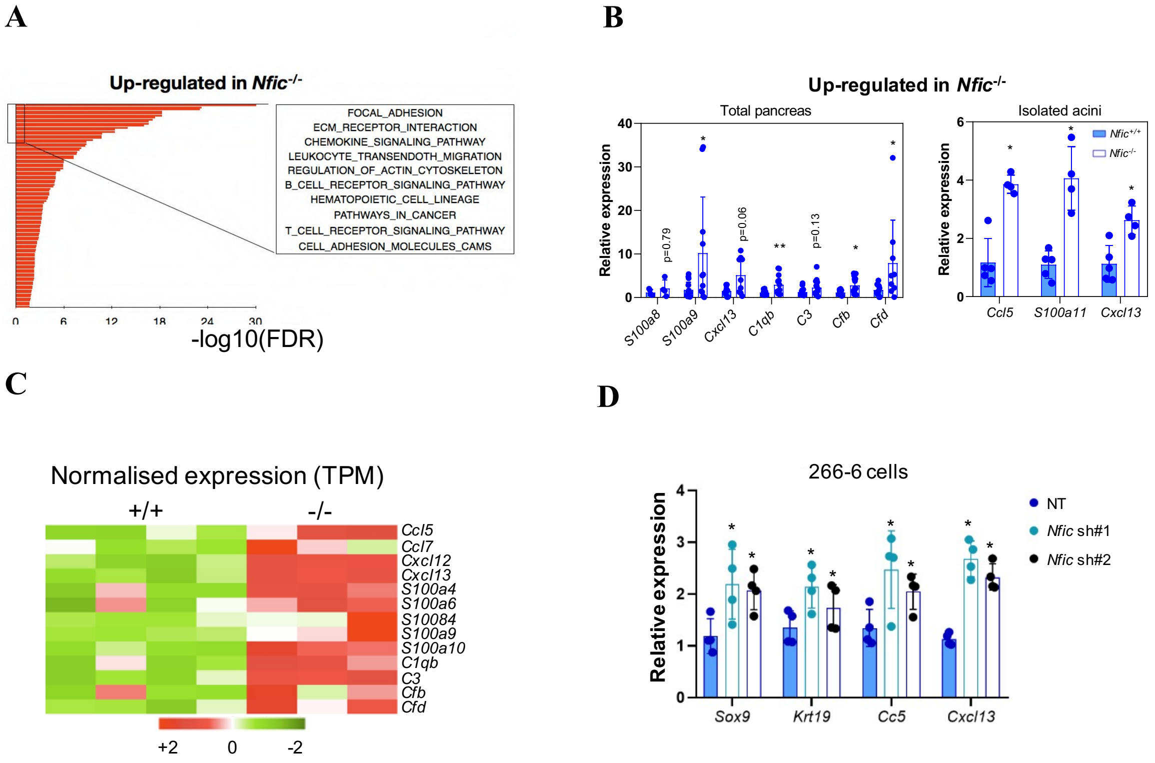

Supplementary Figure 7. NFIC restrains inflammatory gene expression in the mouse pancreas and in 266-6 cells. (A) Gene set enrichment analysis of the pre-ranked list of differentially expressed transcripts in the pancreas of *Nfic*$^{-/-}$ mice compared to wild-type. The pre- ranked list was computed with REACTOME and significant gene sets were ranked by the FDR value. Top-10 most significant are displayed. Up-regulated genes in *Nfic*$^{-/-}$ pancreata belong to several inflammatory gene sets. (B) RT-qPCR of inflammatory transcripts in total pancreas (left panel) or freshly isolated acinar cells (right panel) from wild-type and *Nfic*$^{-/-}$ mice (n$\geq$4 mice/genotype) (*S100a8,* P=0.73; *S100a9,* P= 0.028; *Cxcl13,* P=0.067; *C1qb,* P=0.007; *C3,* P=0.13; *Cfb,* P=0.015; *Cfd* P=0.025). (C) Heatmap showing the expression of transcripts coding for inflammatory genes in WT and *Nfic*$^{-/-}$ pancreata (RNA-Seq data). (D) Lentiviral *Nfic* knockdown in 266-6 cells results in up-regulation of ADM-related and inflammatory genes (n=4 replicates/group). Two-sided Mann-Whitney U test. P<0.1 (#), P<0.05 (*). P<0.01(**). Barplots are presented as mean values +/- SD. Source data are provided as a source data file.

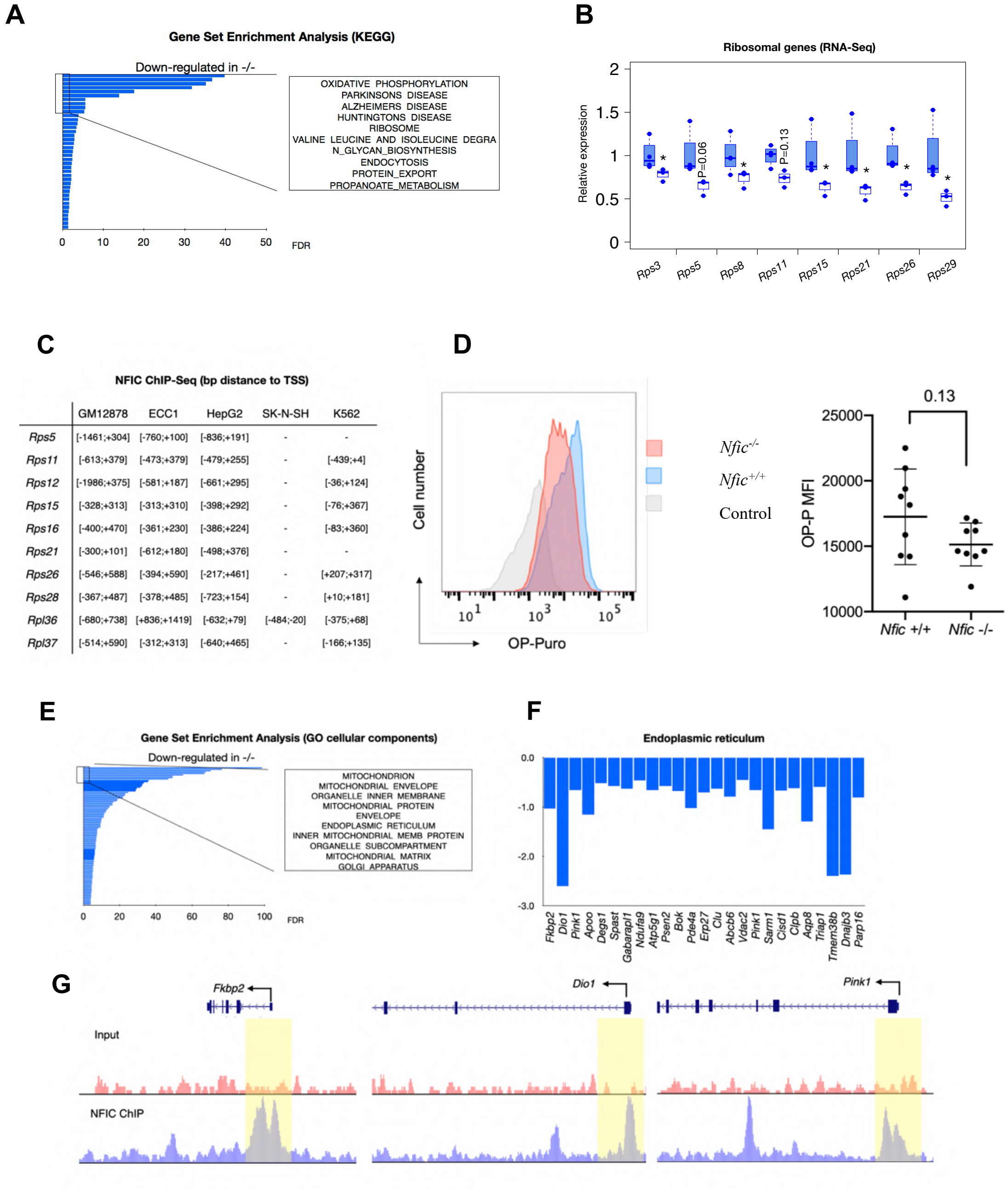

Supplementary Figure 8. NFIC is involved in mRNA and protein metabolism. (A) GSEA of differentially down-regulated genes in *Nfic*$^{-/-}$ pancreata using KEGG. Significant gene sets were ranked by the FDR value and top-10 most significant gene sets are displayed. When multiple gene sets display a similar FDR, a representative gene set is shown (n≥3 mice/genotype). Down-regulated genes in *Nfic*$^{-/-}$ pancreata belong to mRNA, protein metabolism, oxidative phosphorylation, and protein export. (B) Expression analysis by RNA-Seq of ribosomal gene transcripts showing down- regulation in *Nfic*$^{-/-}$ pancreata. Expression values were normalized to those in WT pancreata (n≥3 mice/genotype) (*Rps3,* P= 0.03; *Rps5,* P=0.06; *Rps8,* P= 0.02; *Rps11,* P=0.13; *Rps21,* P=0.04; *Rps26,* P=0.04; *Rps29,* P=0.023; two-tailed Mann-Whitney U-test). (C) NFIC binding to the promoter of genes coding for ribosomal proteins in GM12878, ECC1, HepG2, SK-N-SH, and K562 cells. Data corresponds to distance to the transcription start site (TSS). (D) Fluorescence-based analysis of protein synthesis in freshly isolated pancreatic acini using the Click-It reaction protocol (n=9 mice/genotype). A representative plot corresponding to one WT and one *Nfic*$^{-/-}$ is shown. (E) GSEA of differentially down-regulated genes in *Nfic*$^{-/-}$ pancreata using GO cellular components showing enrichment in mitochondrion, ER, and Golgi complex. (F) Expression analysis by RNA-Seq of genes coding for ER components showing down-regulation in *Nfic*$^{-/-}$ pancreata (FDR<0.05) (n≥3 mice/genotype). (G) Composites of NFIC ChIP-Seq showing a peak at the promoter of *Fkbp2*, *Dio1,* and *Pink1* as representative ER genes. Boxplots are displayed showing the minimum, the maximum, the sample median, and the first and third quartiles. Source data are provided as a source data file.

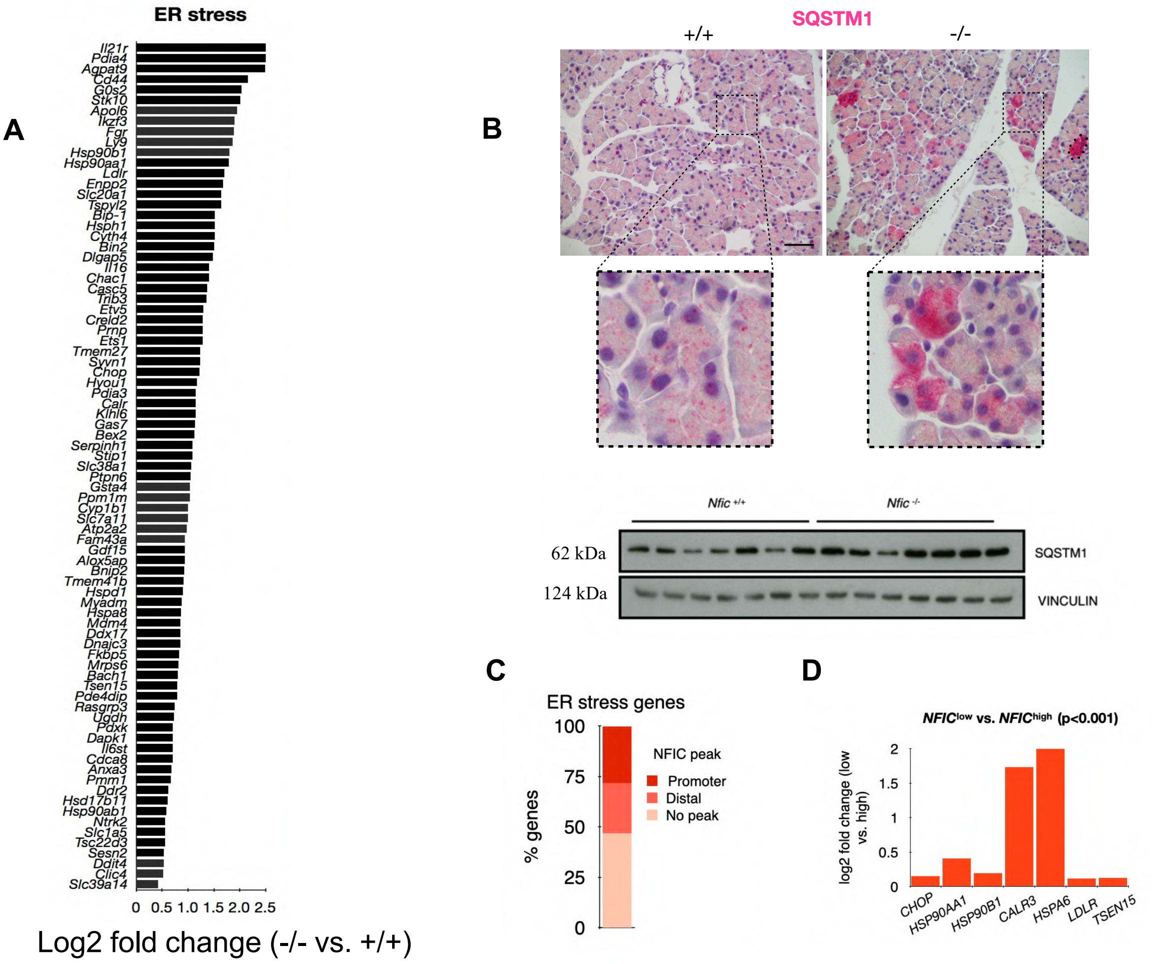

Supplementary Figure 9. NFIC regulates the ER stress response. (A) Expression of genes involved in the UPR and ER stress showing an up-regulation in *Nfic*$^{-/-}$ pancreata (RNA-Seq). (B) Up-regulation of the autophagy marker p62/SQSTM1 in *Nfic*$^{-/-}$ pancreata. IHC shows overexpression in a subset of acinar cells (one representative image from 2 replicates); confirmation by western blotting in the lower panel (n=7 mice/group). (C) Bar graph showing the percentage of ER stress genes with an NFIC peak at the promoter or at distal regions. (D) Expression analysis showing up-regulation of ER stress genes in *NFIC*^{low} compared to *NFIC*^{high} human pancreata (GTEX dataset) (*CHOP,* P=1.7e-5; *HSP90AA1,* P=2.5 e-7; *HSP90B1,* P=1.1 e-5; *CALR3,* P=3.1 e-11; *HSPA,6* P=5.4 e-10; *LDLR,* P=6.1 e-5; *TSEN15,* P= 1.9 e-5; an integration of Fisher's exact test and likelihood ratio were used to calculate statistical significance). Source data are provided as a source data file.

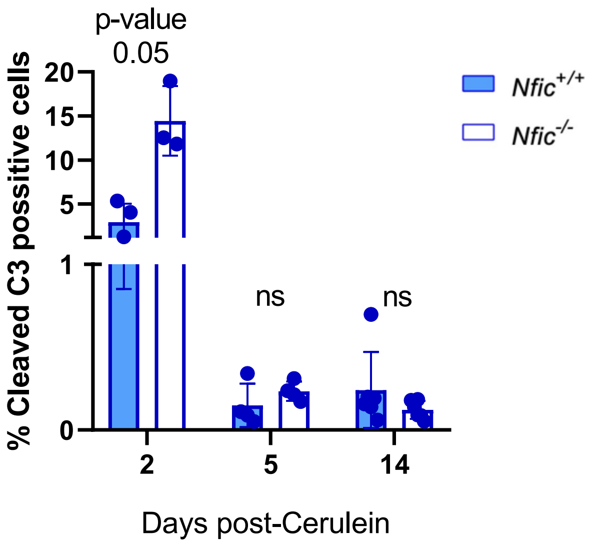

Supplementary Figure 10. Increased cleaved caspase 3 expression 48h post cerulein administration in *Nfic*$^{-/-}$ mice. IHC analysis of cleaved caspase 3 in wild-type and *Nfic*$^{-/-}$ pancreata 48h, 5 days, and 14 days after the induction of pancreatitis showing increased damage in mutant mice (n $\geq$3 mice/genotype). Two-sided Mann-Whitney U test was used to calculate statistical significance. Barplots are presented as mean values +/- SD. Source data are provided as a source data file.

SUPPLEMENTARY TABLES

Supplementary Table 1. List of antibodies used.

Supplementary Table 2. List of primers used for RT-qPCR.

Supplementary Table 3. List of primers used for ChIP-qPCR.

Supplementary Data 1. NR5A2 interactors in normal mouse pancreas tissue identified through IP-MS. Data provided as an Excel file. (One-tailed T-test with a permutation-based FDR control)

Supplementary Data 2. Differentially expressed genes in Nfic-/- vs. WT mouse pancreata. Data provided as an Excel file. (Two-tailed Mann-Whitney U-test)

Supplementary Table 1. List of antibodies used.

Protein	Catalog reference	Working concentration and(technique)
ACTIN	Sigma-Aldrich, MA1-744	0.02 µg/mL (WB)
BIP-1(HSPA5)	Cell Signalling, C50B12	0.8 µg/mL (IF); 0.2 µg/mL (WB)
Long ribosomal RNAs	Thermofisher MA1-13017	2 µg/mL (IF)
CD45	Novus Biologicals, NB110-93609	0.8 µg/mL (IHC)
CDH1	BD transduction, C20 820	0.25-0.35 µg/mL (IHC, IF)
CEL	Abcam, ab87431	0.2 µg/mL (WB)
CHOP (DDIT3)	Cell Signalling, CL63F7	0.2 µg/mL (WB)
Cleaved Caspase-3 (Asp175)	Cell Signalling 9661	1:400 (IHC)
CPA1	RnD Systems, AF2765	1 µg/mL (IF)
CPA1	Biorad (formerly AbD serotec), 1810-0006	0.5 µg/mL (WB)
CTRB1	Biorad (formerly AbD serotec), 2100-0657	0.5 µg/mL (WB)
ERK	Cell Signalling, CST #9102	0.1 µg/mL (WB)
HA- tag	Sigma -Aldrich, F3165	0.1 µg/mL (WB)
Histone H3	Abcam, ab1791	0.05 µg/mL (WB)
IgG (Goat)	Millipore, NI02	
IgG (Mouse)	Santa Cruz, sc-2025	
IgG (Rabbit)	Millipore, 12-370	
INS1	Dako, A0564	1/400 (IF)
KI67	Leica, clone MM1, K2	0.05 µg/mL (IHC)
KI67	Bethyl, IHC-00375	0.05 µg/mL (IHC)
KRT19 (Troma3)	Monoclonal Antibodies Unit (CNIO)	1/25 (IF); 1/50 (IHC)
NFIC	Bethyl, A303-123A	0.4 µg/mL (IHC on formalin-fixed sections)
NFIC	Abcam, ab89516	1.25 µg/mL (IHC/IF on PFA-fixed sections); 0.5 µg/mL (WB); 1 µg/ChIP or IP
NFIC	Abcam, ab245597	1µg/mL (WB)
NR5A2	Everest, EB12283	2 µg/ChIP or IP; 0.5 µg/mL (WB)
P-EIF4E (Ser^{209})	Cell Signalling, CST #9741	0.2 µg/mL (WB)
P-S6 ($Ser^{240/244}$)	Cell Signalling, CST #2215	1 µg/mL (IHC)(IF); 0.2 µg/mL (WB)
P-S6K1(Thr^{389})	Cell Signalling, CST #9205	0.2 µg/mL (WB)
PTF1A	Kindly provided by B. Bréant (INSERM)	1/400 (IHC); 1/200 (IF); 1/1000 (WB); 1/500 (ChIP)
SOX9	Millipore AB535	0.4 µg/mL (IF)
VINCULIN	Sigma -Aldrich, Clone hVIN-1	0.1-0.13 µg/mL (WB)

Supplementary Table 2. List of primers used for RT-qPCR.

Gene	Forward	Reverse
Amy2a5	TGGCGTCAAATCAGGAACATG	AAAGTGGCTGACAAAGCCCAG
Bip1/Hspa5	TCATCGGACGCACTTGGAA	CAACCACCTTGAATGGCAAGA
Cel	AAGTTGCCCGTGAAAAAGCAG	ATGGTAGCAAATAGGTGGCCG
Cela1	TGTGTCACACCCCTACTGGA	TTGTTAGCCAGGATGGTTCC
Cela2a	AGGTGGAGGATGATGTGAGC	TGTCAGAACCCAGTTGTTGG
Cela3b	AGTTGTCAATGGCGAGGAAG	CAGAACCCAGTCAGGGGTAA
C1qb	CACAGAACACCAGGATTCCA	CCCACTGTGTCTTCATCAGC
Ccl5	GCCTCACCATATGGCTCGGACA	CCTTGACGTGGGCACGAGGC
Ccl5	GTGCCCACGTCAAGGAGTAT	CCCACTTCTTCTCTGGGTTG
Ccl7	CCAACCAGATGGGCCCAATGC ATCC	TCAGCGCAGACTTCCATGCCC
Ccl9	TGGGCCCAGATCACACATGCAA C	CGGCCTGGTACACCCACCAC
Complement C3 (C3)	AGTGCTACTGCTGCTGTTGG	GCCGTAGGACATTGGGAGTA
Complement Factor B (CFB)	CCGAGACCAAAAGATTGTCC	TCCCCATTTTCAAAGTCCTG
Complement Factor D (CFD)	TGCACAGCTCCGTGTACTTC	CTCCTGGCCACCCAGAAT
Complement Factor P (CFP)	TATGCCTTCCAGGAGCATGA	CCATAAGGACCATGCTGACC
Chop/Ddit3	GTCCCTAGCTTGGCTGACAGA	TGGAGAGCGAGGGCTTTG
Cpa1	TACACCCACAAAACGAATCGC	GCCACGGTAAGTTTCTGAGCA
Ctrb1	GCAAGACCAAATACAATGCCC	TGCGCAGATCATCACATCG
Cxcl13	GCCTCTCTCCAGGCCACGGTAT	AGCCATTCCCAGGGGGCGTA
Hprt	CGTCGTGATTAGCGATGATGA	ACAATGTGATGGCCTCCCA
Hsd17b11	CTGACTGCCTACGAATTTGCC	GTTTCCTCGATGCCGTTCTTATT
Nfic	TGGACCTGTACCTGGCCTAC	GCTCTCCTGGAAGTCTGTGG
Nr5a2	CGATCAGCGGGAGTTTGTAT	CATTCACCTGCTCTTGGACA
Nr5a2	GTTGAGTGGGCCAGGAGTAGTA	ACGCGACTTCTGTGTGTGAG
Nr5a2	TTGAGTGGGCCAGGAGTAGTA	ACGCGACTTCTGTGTGTGAG
Nr5a2	TCATGCTGCCCAAAGTGGAGA	TGGTTTTGGACAGTTCGCTT
Pnlip	ACAGATCAACACCCGCTTTC	CGGGTTTTTCTGTTTGTTCG
Ptf1a	AACCAGGCCCAGAAGGTTAT	AAAGAGAGTGCCCTGCAAGA
Rnase1	CAGCAGGACAAACAATGGAA	CCAATTCGTCTTGGAGTTCA
Spink3	GGCAACTAGCCTCTTTTCCA	GACAATGAAGGTGGCTGTCA
Nr0b2	AGCTGGGTCCCAAGGAGTAT	AGTGAGCCTCCTGTTGCAG
Rps5	CAAGCTCTTTGGGAAATGGA	GGGCAGGTACTTGGCATACT
Rps8	TTAGAAACCGGACCGTGAAG	TCTCAGGGCACGGTACTTCT
Rps28	CTCCTCTCCGCCAGATCG	GCCTTTGACATTTCGGATGA
S100a10	TCTTCGGCACTAGCCTCATC	ATTTGGGATGGCATTTTGAA
S100a11	CAAAAGTACAGCGGGAAGGA	CTTCTTCATCATGCGGTCAA
Spliced Xbp1	AAGAACACGCTTGGGAATGG	CTGCACCTGCTGCGGAC
45S	GAGCTGGTGGTGGCGCTCC	CTTGCCCCTCCTTCTCT
Mature 18S	GATGGTAGTCGCCGTGCC	GCCTGCTGCCTTCCTTGG
Mature 5.8S	ACTCGGCTCGTGCGTC	CCGACGCTCAGACAGG
Mature 28S	GACGCGCATGAATGGA	TGTGGTTTCGCTGGATAGTAGGT
5.8S 5' Junction	TACGACTCTTAGCGGTGGATCA	TCACATTAATTCTCGCAGCTAGCT
28S 5' Junction	CTGACCGCGACCTCAGAT	TCCGCTGACTAATATGCTTAAATTC A

Supplementary Table 3. List of primers used for ChIP-qPCR.

Gene (promoter)	Forward	Reverse
Cel	CCATAAATACTGGAGAAGAGGAGAGAA	CAGGCGCCCCATGGT
Cela2a	GGCACTCTTCTCAGGGTGTT	GAGCATGTGTGTCCTTCCATT
Cela2a	GGACCTGTCTTTGGCATGTT	TTTCCATTCCCTTGTCGTTC
Cpa1	CATGGTCAAGGGTGAAAGC	GAGGCAGGAGTTCCAAACTG
Cpa1	AGCTGACCCCATGGTCAAGGG	GGGTCCCTGGGGACAGTTCCC
Ctrb1	CCAACCAGAAAGGTCCAAGA	CAGAGCAGCTGTCCTTTTCC
Chop/Ddit3	CCTCCCACCACCATCGAC	GAGGAGGTGAGTGAGTCATGC
Hspa5/Bip-1	TGTTTCTCCTTCACCCCAAG	GCGGAGAAAGGGAATAGGTT
Hsp90aa1	TACGCAACCAGAACCCCAGT	AGGCAAAAGGTCCCCCTTC
Negative region	TTGGGTGTTGGGAACTGAAT	CCCTTCTCTGCCTTCTGATG
Nfic promoter #1	AGGCTGCTAGCGCTGTTCTA	CACTGCCAAAGATGGAGGAG
Nfic promoter #2	CACCTGGCTGTCATGTGTC	CAATTGCATGTCAGGCTTGT
Nfic promoter #3	GGAGGAGGGGAGTGAGAGTT	ACACGACTCCTGCCTGTCTC
Nr0b2	GACAAGCTGACAGTCACACACTAGAA	GCCCTGGCACCTGGTTTA
Pnlip	CCGGTACTTCTCTGGGCTTA	CTACGGACAACGAGCAAACA
Pnlip	CGCTCAGCCTCCGGTACTT	GGCTCCAAGGACGTACTTCTAATT
Rps5	TTAAGGGTGCTGATTGTAACGA	AGTATTTTGAGACAAAGTCCGATT
Rps8	CTGTGAACAGAGCGTTGGAG	AGGCCAACCTGGTCTACAAA
Rps28	GGGAAGGATTTGGGTTCTGT	GGAGCTAAAGCCAGGCAATC

Source data of Supplementary blots

Supplementary Figure 1A

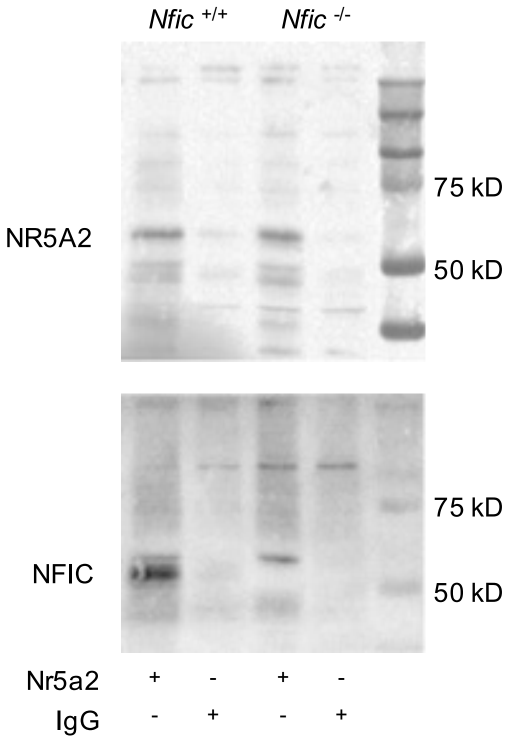

Supplementary Figure 9B

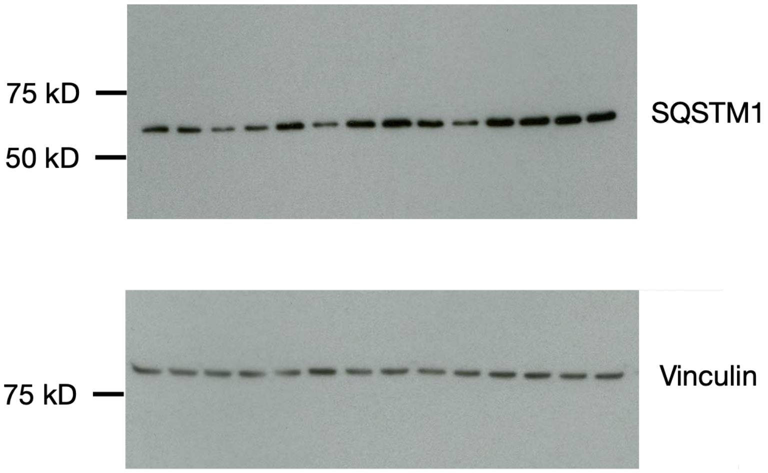

Supplement: Supplementary file 1 — Supplementary Information [file 41467_2023_39291_MOESM1_ESM.pdf]
