# Supplementary material for: NFIC regulates ribosomal biology and ER stress in pancreatic acinar cells and restrains PDAC initiation
Source: Nat Commun. 2023 Jun 23;14:3761. doi: 10.1038/s41467-023-39291-x (PMC10290102; doi:10.1038/s41467-023-39291-x)
Supplement: Supplementary file 3 — Description of Additional Supplementary Files [file 41467_2023_39291_MOESM3_ESM.pdf]

Description of Additional Supplementary Files

File Name: Supplementary Data 1
Description: **NR5A2 interactors in normal mouse pancreas tissue identified through IP-MS. Data provided as an Excel file.** (One-tailed T-test with a permutation-based FDR control)

File Name: Supplementary Data 2
Description: **Differentially expressed genes in Nfic-/- vs. WT mouse pancreata. Data provided as an Excel file.** (Two-tailed Mann-Whitney U-test)
