# Supplementary material for: NFIC regulates ribosomal biology and ER stress in pancreatic acinar cells and restrains PDAC initiation
Source: Nat Commun. 2023 Jun 23;14:3761. doi: 10.1038/s41467-023-39291-x (PMC10290102; doi:10.1038/s41467-023-39291-x)
Supplement: Supplementary file 6 — Reporting Summary [file 41467_2023_39291_MOESM6_ESM.pdf]

nature portfolio

Corresponding author(s): Francisco X Real

Last updated by author(s): May29,2023

Reporting Summary

Nature Portfolio wishes to improve the reproducibility of the work that we publish. This form provides structure for consistency and transparency in reporting. For further information on Nature Portfolio policies, see our Editorial Policies and the Editorial Policy Checklist.

Statistics

For all statistical analyses, confirm that the following items are present in the figure legend, table legend, main text, or Methods section.

n/a	Confirmed	
☐	☒	The exact sample size (*n*) for each experimental group/condition, given as a discrete number and unit of measurement
☐	☒	A statement on whether measurements were taken from distinct samples or whether the same sample was measured repeatedly
☐	☒	The statistical test(s) used AND whether they are one- or two-sided<br>*Only common tests should be described solely by name; describe more complex techniques in the Methods section.*
☐	☒	A description of all covariates tested
☐	☒	A description of any assumptions or corrections, such as tests of normality and adjustment for multiple comparisons
☐	☒	A full description of the statistical parameters including central tendency (e.g. means) or other basic estimates (e.g. regression coefficient) AND variation (e.g. standard deviation) or associated estimates of uncertainty (e.g. confidence intervals)
☐	☒	For null hypothesis testing, the test statistic (e.g. *F*, *t*, *r*) with confidence intervals, effect sizes, degrees of freedom and *P* value noted<br>*Give P values as exact values whenever suitable.*
☒	☐	For Bayesian analysis, information on the choice of priors and Markov chain Monte Carlo settings
☒	☐	For hierarchical and complex designs, identification of the appropriate level for tests and full reporting of outcomes
☒	☐	Estimates of effect sizes (e.g. Cohen's *d*, Pearson's *r*), indicating how they were calculated

Our web collection on statistics for biologists contains articles on many of the points above.

Software and code

Policy information about availability of computer code

Data collection	RNA-Seq of human pancreas samples were downloaded from GTEX (https://gtexportal.org/home/datasets, version 6).<br>Principal component analysis and boxplot were performed using R (https://www.r-project.org/)<br>Gene Set Enrichment analysis was performed using the molecular signature database of GSEA (http://www.gsea-msigdb.org/gsea/msigdb/annotate.jsp).<br>NR5A2 ChIP-Seq in adult pancreata (SRR389293, SRR389294), NR5A2 ChIP-Seq in ES cells (GSM470523, GSM470524), PTF1A ChIP-Seq in adult pancreata (GSM2051452, GSM2051453), MIST1 ChIP-Seq in adult pancreata (GSM2299654,GSM2299654, GSM2299655) were downloaded from the Gene Expression Omnibus website (https://www.ncbi.nlm.nih.gov/geo/)<br>NFIC ChIP-Seq data using GM12878, ECC1, HepG2, SK-N-SH and K562 cells were downloaded from (https://www.encodeproject.org/targets/NFIC-human/)
Data analysis	Differential expression analysis for RNA-Seq samples of human pancreas samples using the DEGseq package of R (https://bioconductor.org/packages/release/bioc/html/DEGseq.html) using P<0.001 as cutoff for significance.<br>RNA-Seq analysis of mouse pancreas samples was performed using Nextpresso (http://ubio.bioinfo.cnio.es/people/ograna/nextpresso/)<br>ChIP-Seq data was performed using RUbioSeq+ pipeline (Rubio-Camarillo et al., 2017). Merging of replicate peaks and peak annotation was done using HOMER. Peak calling, annotation and motif enrichment was identified using HOMER (Heinz et al., 2010; http://homer.ucsd.edu/homer/).

For manuscripts utilizing custom algorithms or software that are central to the research but not yet described in published literature, software must be made available to editors and reviewers. We strongly encourage code deposition in a community repository (e.g. GitHub). See the Nature Portfolio guidelines for submitting code & software for further information.

Data

Policy information about availability of data

All manuscripts must include a data availability statement. This statement should provide the following information, where applicable:

- Accession codes, unique identifiers, or web links for publicly available datasets
- A description of any restrictions on data availability
- For clinical datasets or third party data, please ensure that the statement adheres to our policy

Genome-wide data generated in this study has been uploaded to Gene Expression Omnibus (GEO) and will be available upon acceptance of the manuscript. Links to publicly available datasets are provided in the Material and Method section of the manuscript and in this reporting summary form.

Field-specific reporting

Please select the one below that is the best fit for your research. If you are not sure, read the appropriate sections before making your selection.

☒ Life sciences ☐ Behavioural & social sciences ☐ Ecological, evolutionary & environmental sciences

For a reference copy of the document with all sections, see nature.com/documents/nr-reporting-summary-flat.pdf

Life sciences study design

All studies must disclose on these points even when the disclosure is negative.

Sample size	No previous sample size calculation was used. The number of mice used was based on the previous experience of the laboratory in using these assays and mouse strains. In selected cases, a pilot experiment was performed to decide the final number of mice to be used. For selected experiments (i.e. RNA-Seq) a "feasible" number of mice was used that was estimated to provide sufficient statistical power.
Data exclusions	No data were excluded from the analysis except for data from one Nfic-/- mouse used for the RNA-Seq analysis in which histology of the pancreas differed significantly from that of the other three mice and from other mice of the same genotype used in additional experiments. Among the two replicates of each NFIC ChIP-Seq in cell lines, the one with highest number of identified target genes was taken: replicate 1 of NFIC ChIP-Seq in GM12878, NFIC ChIP-Seq in HepG2 and NFIC ChIP-Seq in SK-N-SH and replicate 2 of NFIC ChIP-Seq in ECC1 cell line.
Replication	For all major experiments, at least two and generally 3 independent experiments were performed.
Randomization	Samples were allocated to experimental group (a randomization method was not used) without any previous selection, as stated in the text.
Blinding	Samples were coded. Experiments were performed and then data were decoded. The code was unknown to the investigator at the time of analysis. All histological analyses were performed with blind assessment of the samples.

Reporting for specific materials, systems and methods

We require information from authors about some types of materials, experimental systems and methods used in many studies. Here, indicate whether each material, system or method listed is relevant to your study. If you are not sure if a list item applies to your research, read the appropriate section before selecting a response.

Materials & experimental systems

n/a	Involved in the study	
☐	☒	Antibodies
☐	☒	Eukaryotic cell lines
☒	☐	Palaeontology and archaeology
☐	☒	Animals and other organisms
☐	☒	Human research participants
☐	☒	Clinical data
☒	☐	Dual use research of concern

Methods

n/a	Involved in the study	
☐	☒	ChIP-seq
☐	☐	Flow cytometry
☐	☐	MRI-based neuroimaging

Antibodies

Antibodies used	A list with all antibodies used and antibody source and concentration is provided in the Methods section and in Supplementary Material. The following antibodies were used:<br><br>ACTIN. Sigma-Aldrich, MA1-744 0.02 µg/mL (WB).<br>BIP-1(HSPA5). Cell Signalling, C50B12 0.8 µg/mL (IF); 0.2 µg/mL (WB).<br>Long ribosomal RNAs. Thermofisher MA1-13017. 2 µg/mL (IF).

	CD45. Novus Biologicals, NB110-93609 0.8 µg/mL (IHC).<br>CDH1. BD transduction, C20 820. 0.25-0.35 µg/mL (IHC,IF).<br>CEL. Abcam, ab87431 0.2 µg/mL (WB).<br>CHOP. (DDIT3) Cell Signalling, CL63F7. 0.2 µg/mL (WB).<br>CPA1. RnD Systems, AF2765 1 µg/mL (IF).<br>CPA1 . Biorad (formerly AbD serotec), 1810-0006. 0.5 µg/mL (WB).<br>CTRB1. Biorad (formerly AbD serotec), 2100-0657. 0.5 µg/mL (WB).<br>ERK. Cell Signalling, CST #9102 0.1 µg/mL (WB).<br>HA- tag. Sigma -Aldrich, F3165 0.1 µg/mL (WB).<br>Histone H3. Abcam, ab1791 0.05 µg/mL (WB).<br>IgG (Goat). Millipore, NI02.<br>IgG (Mouse). Santa Cruz, sc-2025.<br>IgG (Rabbit). Millipore, 12-370.<br>INS1. Dako, A0564 1/400 (IF).<br>KI67. Leica, clone MM1, K2 0.05 µg/mL (IHC).<br>KI67. Bethyl. IHC-00375 0.05 µg/mL (IHC).<br>KRT19. Troma3. Monoclonal Antibodies Unit, CNIO. " 1/25 (IF); 1/50 (IHC).<br>NFIC. Bethyl. A303-123A 0.4 µg/mL (IHC on formalin-fixed sections).<br>NFIC. Abcam, ab89516. 1.25 µg/mL (IHC/IF on PFA-fixed sections); 0.5 µg/mL (WB); 1 µg/ChIP or IP.<br>NR5A2. Everest, EB12283. 2 µg/ChIP or IP; 0.5 µg/mL (WB).<br>P-EIF4E (Ser209). Cell Signalling, CST #9741 0.2 µg/mL (WB).<br>P-S6 (Ser240/244). Cell Signalling, CST #2215 1 µg/mL (IHC)(IF); 0.2 µg/mL (WB).<br>P-S6K1(Thr389). Cell Signalling, CST #9205 0.2 µg/mL (WB).<br>PTF1A Kindly provided by B. Bréant (INSERM). 1/400 (IHC); 1/200 (IF); 1/1000 (WB); 1/500 (ChIP).<br>SOX9. Millipore AB535 0.4 µg/mL (IF).<br>VINCULIN. Sigma -Aldrich, Clone hVIN-1 0.1-0.13 µg/mL (WB).
Validation	Validation was performed following variable strategies: cellular distribution, size of bands in western blotting experiments, for critical antibodies knockout cells/tissues were used for validation (i.e. NFIC antibody using NFIC knockdown in 266-6 cells and NFIC-/- pancreata).

Eukaryotic cell lines

Policy information about cell lines

Cell line source(s)	HEK293 cells (from ATCC) and 266-6 (from I. Rooman (VUB, Brussels) who obtained them from ATCC).
Authentication	HEK293 cells were not authenticated as they came from ATCC. 266-6 cells are known to the investigators and unique and experimental analyses showed that they are the expected cells (only one mouse cell line with acinar features is available to our knowledge worldwide).
Mycoplasma contamination	Yes, tested and only Mycoplasma-free cultures were used.
Commonly misidentified lines<br>(See ICLAC register)	No commonly misidentified cell lines were used.

Animals and other organisms

Policy information about studies involving animals; ARRIVE guidelines recommended for reporting animal research

Laboratory animals	Information provided in detail in the Methods section. The Nfic-/- strains of Mus muscules was validated using specific anti-NFIC antibodies.<br>Source of mice and references:<br>Ptf1a-Cre - Kawaguchi, Y., et al. The role of the transcriptional regulator Ptf1a in converting intestinal to pancreatic progenitors. Nat. Genet. 32, 128-134 (2002).<br>Nfic knock-out: Steele-Perkins G, Butz KG, et al. Essential role for NFI-C/CTF transcription-replication factor in tooth root development. Mol Cell Biol 2003; 23:1075-84.<br>KrasG12V: Guerra C, Schuhmacher AJ, et al. Chronic pancreatitis is essential for induction of pancreatic ductal adenocarcinoma by K-Ras oncogenes in adult mice. Cancer Cell 2007; 11:291-302
Wild animals	*Provide details on animals observed in or captured in the field; report species, sex and age where possible. Describe how animals were caught and transported and what happened to captive animals after the study (if killed, explain why and describe method; if released, say where and when) OR state that the study did not involve wild animals.*
Field-collected samples	*For laboratory work with field-collected samples, describe all relevant parameters such as housing, maintenance, temperature, photoperiod and end-of-experiment protocol OR state that the study did not involve samples collected from the field.*
Ethics oversight	All animal procedures were approved by local and regional ethics committees (Institutional Animal Care and Use Committee and Ethics Committee for Research and Animal Welfare, Instituto de Salud Carlos III) and performed according to the European Union guidelines.

Note that full information on the approval of the study protocol must also be provided in the manuscript.

Human research participants

Policy information about studies involving human research participants

Population characteristics	*Describe the covariate-relevant population characteristics of the human research participants (e.g. age, gender, genotypic information, past and current diagnosis and treatment categories). If you filled out the behavioural & social sciences study design questions and have nothing to add here, write "See above."*
Recruitment	*Describe how participants were recruited. Outline any potential self-selection bias or other biases that may be present and how these are likely to impact results.*
Ethics oversight	*Identify the organization(s) that approved the study protocol.*

Note that full information on the approval of the study protocol must also be provided in the manuscript.

Clinical data

Policy information about clinical studies

All manuscripts should comply with the ICMJE guidelines for publication of clinical research and a completed CONSORT checklist must be included with all submissions.

Clinical trial registration	*Provide the trial registration number from ClinicalTrials.gov or an equivalent agency.*
Study protocol	*Note where the full trial protocol can be accessed OR if not available, explain why.*
Data collection	*Describe the settings and locales of data collection, noting the time periods of recruitment and data collection.*
Outcomes	*Describe how you pre-defined primary and secondary outcome measures and how you assessed these measures.*

ChIP-seq

Data deposition

☒ Confirm that both raw and final processed data have been deposited in a public database such as GEO.

☒ Confirm that you have deposited or provided access to graph files (e.g. BED files) for the called peaks.

Data access links<br>*May remain private before publication.*	RNA sequencing data have been deposited in GEO with accession number GSE126907 and NFIC ChIP sequencing data have been deposited in GEO with accession number GSE181098
Files in database submission	NFIC ChIP-Seq:<br>ChIP-NFIC_33188_AGTCAA.fastq.gz (NFIC ChIP replicate 1)<br>NFIC-ChIP2_35112_GTTTCG.fastq.gz (NFIC ChIP replicate 2)<br>NFIC-ChIP5_35112_CGTACG.fastq.gz (NFIC ChIP replicate 3)<br>Input-Panc-INRT_33188_GGCTAC.fastq.gz (input DNA)<br>peaksannotation.txt<br><br>RNA-Seq NFIC-/- pancreata<br>genes.count_table<br>genes.fpkm_table<br>metadata_spreadsheet.xls<br>PUB22_1.fastq.gz (Nfic+/+ replicate 1)<br>PUB33_1.fastq.gz (Nfic+/+ replicate 2)<br>PUB48_1.fastq.gz (Nfic+/+ replicate 3)<br>PUB49_1.fastq.gz (Nfic+/+ replicate 4)<br>PUB23_1.fastq.gz (Nfic-/- replicate 1)<br>PUB24_1.fastq.gz (Nfic-/- replicate 2)<br>PUB38_1.fastq.gz (Nfic-/- replicate 3)
Genome browser session<br>(e.g. UCSC)	http://homer.ucsd.edu/hubs//NFICChIPSeq/hub.txt

Methodology

Replicates	For NFIC ChIP-Seq, three wild type mouse pancreata were used. One input file was used to normalize ChIP enrichment.<br>For RNA-Seq, 4 Nfic+/+ and 3 Nfic-/- pancreata were used.
Sequencing depth	For NFIC ChIP-Seq, 17,636061 reads were obtained for the input and 50,208085 reads were obtained for the combined three ChIP-Seq.<br>For RNA-Seq, > 32 million reads were obtained for each sample.

Antibodies	NFIC. Abcam, ab89516.
Peak calling parameters	For NFIC ChIP-Seq,<br>Alignment performed using BWA aligner using mm10 as reference genome<br>Duplicates marked and removed using Picard<br>Normalization performed with MACS2<br>Peak calling was performed using findPeaks tool of HOMER with default parameters.
Data quality	Only peaks with log2 fold change > 1 and FDR < 0.05 over input DNA were used for downstream analysis
Software	Peak calling was performed using findPeaks tool of HOMER with default parameters.

Flow Cytometry

Plots

Confirm that:

☐ The axis labels state the marker and fluorochrome used (e.g. CD4-FITC).

☐ The axis scales are clearly visible. Include numbers along axes only for bottom left plot of group (a 'group' is an analysis of identical markers).

☐ All plots are contour plots with outliers or pseudocolor plots.

☐ A numerical value for number of cells or percentage (with statistics) is provided.

Methodology

Sample preparation	*Describe the sample preparation, detailing the biological source of the cells and any tissue processing steps used.*
Instrument	*Identify the instrument used for data collection, specifying make and model number.*
Software	*Describe the software used to collect and analyze the flow cytometry data. For custom code that has been deposited into a community repository, provide accession details.*
Cell population abundance	*Describe the abundance of the relevant cell populations within post-sort fractions, providing details on the purity of the samples and how it was determined.*
Gating strategy	*Describe the gating strategy used for all relevant experiments, specifying the preliminary FSC/SSC gates of the starting cell population, indicating where boundaries between "positive" and "negative" staining cell populations are defined.*

☐ Tick this box to confirm that a figure exemplifying the gating strategy is provided in the Supplementary Information.

Magnetic resonance imaging

Experimental design

Design type	*Indicate task or resting state; event-related or block design.*
Design specifications	*Specify the number of blocks, trials or experimental units per session and/or subject, and specify the length of each trial or block (if trials are blocked) and interval between trials.*
Behavioral performance measures	*State number and/or type of variables recorded (e.g. correct button press, response time) and what statistics were used to establish that the subjects were performing the task as expected (e.g. mean, range, and/or standard deviation across subjects).*

Acquisition

Imaging type(s)	*Specify: functional, structural, diffusion, perfusion.*
Field strength	*Specify in Tesla*
Sequence & imaging parameters	*Specify the pulse sequence type (gradient echo, spin echo, etc.), imaging type (EPI, spiral, etc.), field of view, matrix size, slice thickness, orientation and TE/TR/flip angle.*
Area of acquisition	*State whether a whole brain scan was used OR define the area of acquisition, describing how the region was determined.*

Diffusion MRI ☐ Used ☐ Not used

Preprocessing

Preprocessing software	*Provide detail on software version and revision number and on specific parameters (model/functions, brain extraction, segmentation, smoothing kernel size, etc.).*
Normalization	*If data were normalized/standardized, describe the approach(es): specify linear or non-linear and define image types used for transformation OR indicate that data were not normalized and explain rationale for lack of normalization.*
Normalization template	*Describe the template used for normalization/transformation, specifying subject space or group standardized space (e.g. original Talairach, MNI305, ICBM152) OR indicate that the data were not normalized.*
Noise and artifact removal	*Describe your procedure(s) for artifact and structured noise removal, specifying motion parameters, tissue signals and physiological signals (heart rate, respiration).*
Volume censoring	*Define your software and/or method and criteria for volume censoring, and state the extent of such censoring.*

Statistical modeling & inference

Model type and settings	*Specify type (mass univariate, multivariate, RSA, predictive, etc.) and describe essential details of the model at the first and second levels (e.g. fixed, random or mixed effects; drift or auto-correlation).*
Effect(s) tested	*Define precise effect in terms of the task or stimulus conditions instead of psychological concepts and indicate whether ANOVA or factorial designs were used.*

Specify type of analysis: ☐ Whole brain ☐ ROI-based ☐ Both

Statistic type for inference (See Eklund et al. 2016)	*Specify voxel-wise or cluster-wise and report all relevant parameters for cluster-wise methods.*
Correction	*Describe the type of correction and how it is obtained for multiple comparisons (e.g. FWE, FDR, permutation or Monte Carlo).*

Models & analysis

n/a	Involved in the study
☐	☐ Functional and/or effective connectivity
☐	☐ Graph analysis
☐	☐ Multivariate modeling or predictive analysis

Functional and/or effective connectivity	*Report the measures of dependence used and the model details (e.g. Pearson correlation, partial correlation, mutual information).*
Graph analysis	*Report the dependent variable and connectivity measure, specifying weighted graph or binarized graph, subject- or group-level, and the global and/or node summaries used (e.g. clustering coefficient, efficiency, etc.).*
Multivariate modeling and predictive analysis	*Specify independent variables, features extraction and dimension reduction, model, training and evaluation metrics.*
