# Supplementary material for: NFIC regulates ribosomal biology and ER stress in pancreatic acinar cells and restrains PDAC initiation
Source: Nat Commun. 2023 Jun 23;14:3761. doi: 10.1038/s41467-023-39291-x (PMC10290102; doi:10.1038/s41467-023-39291-x)
Supplement: Supplementary file 7 — Source Data [file 41467_2023_39291_MOESM7_ESM.zip › WB_Files.pdf]

Figure 1D

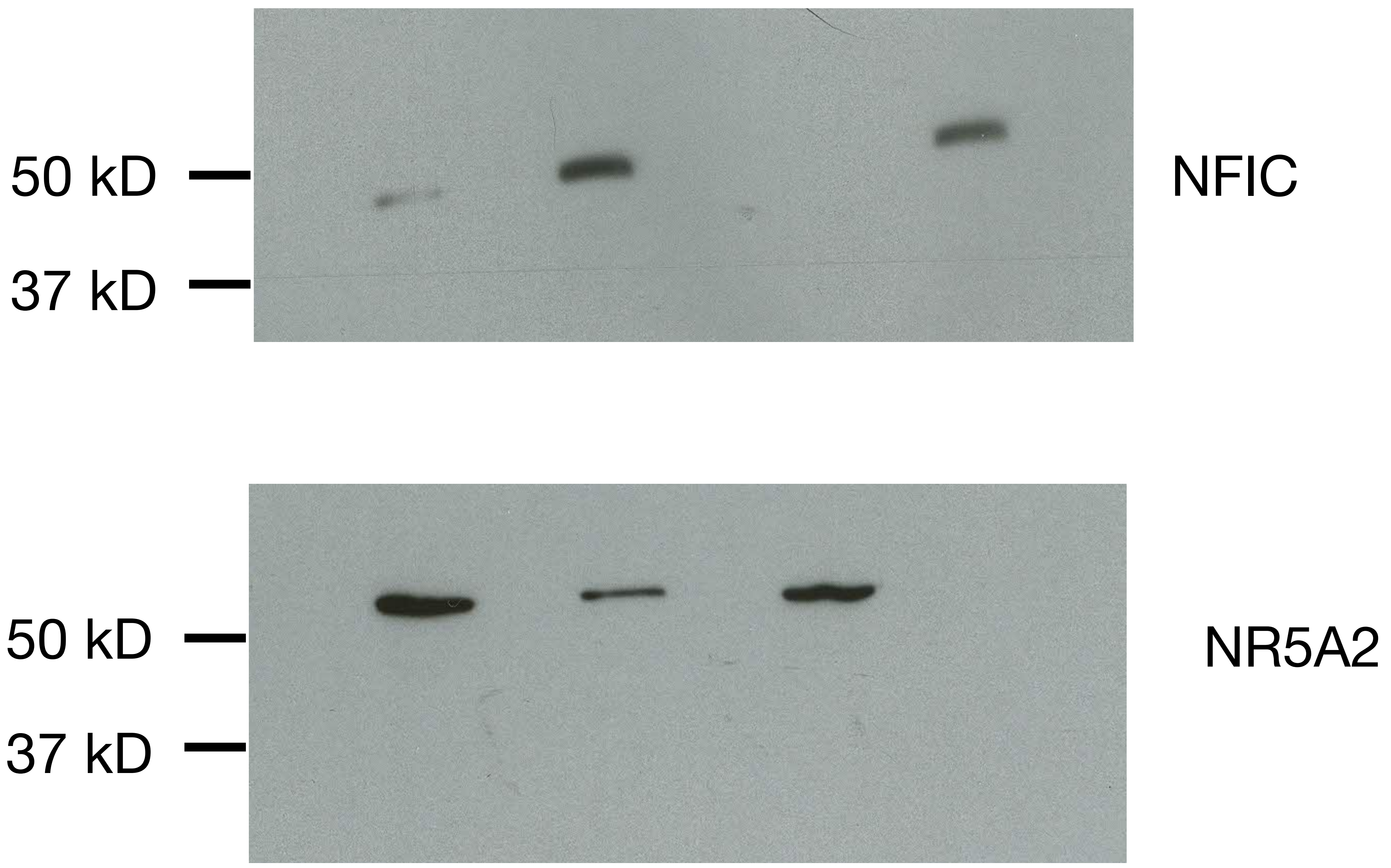

Figure 1I

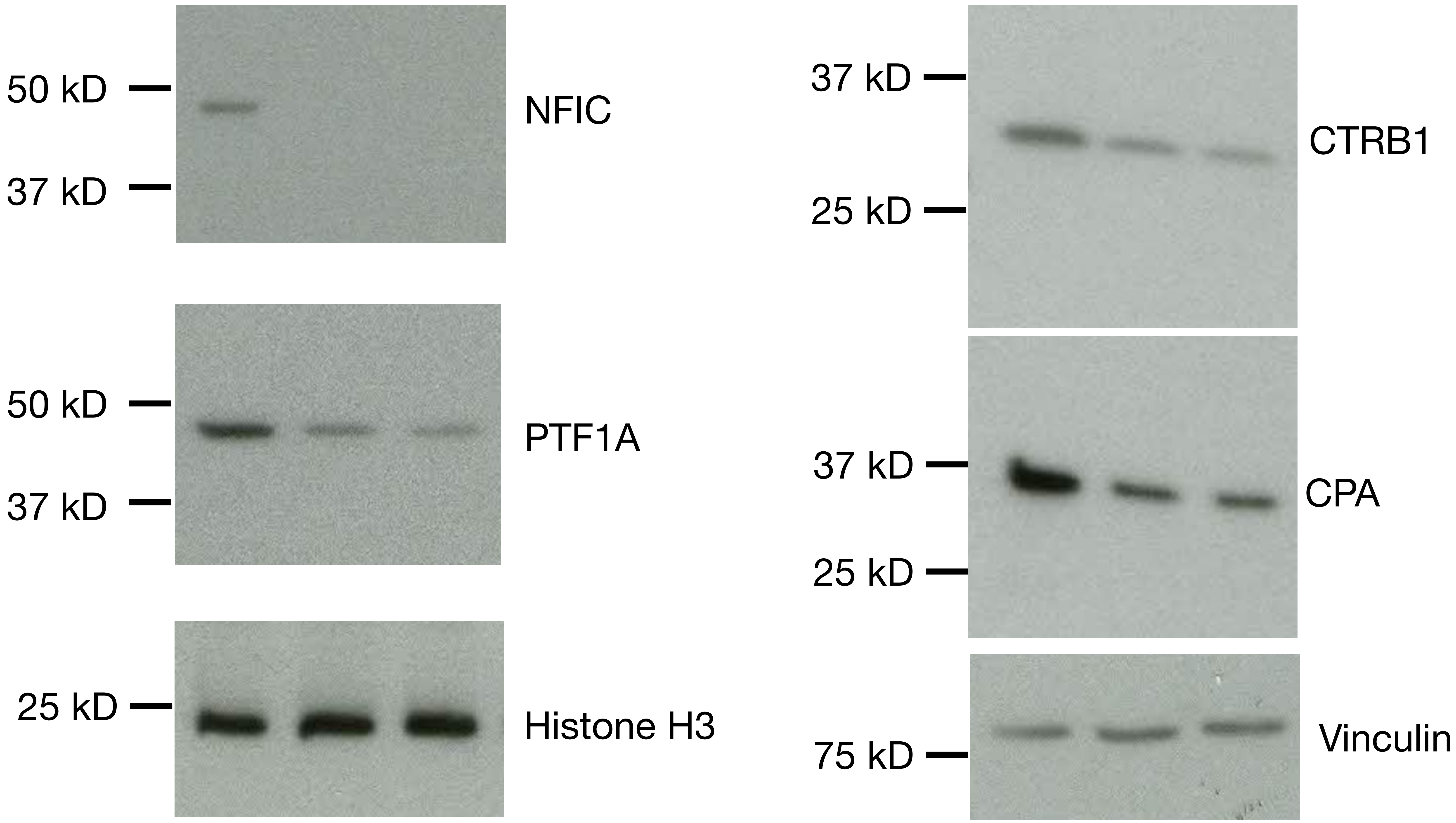
50 kD
37 kD
NFIC
50 kD
37 kD
PTF1A
25 kD
Histone H3
37 kD
25 kD
CTRB1
37 kD
25 kD
CPA
75 kD
Vinculin

Figure 2D

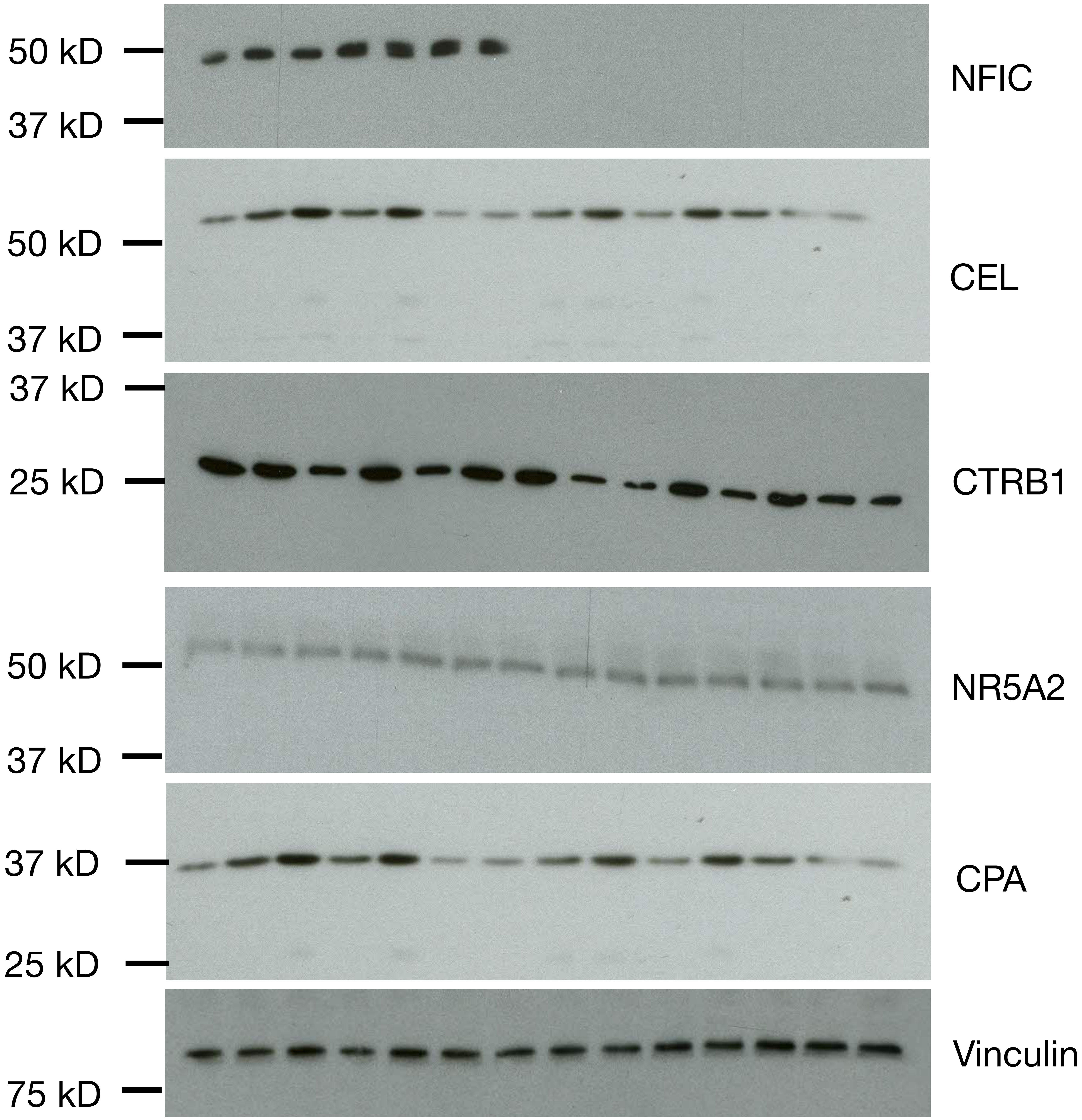
50 kD
37 kD
NFIC
50 kD
37 kD
CEL
37 kD
25 kD
CTRB1
50 kD
37 kD
NR5A2
37 kD
25 kD
CPA
75 kD
Vinculin

Figure 5D

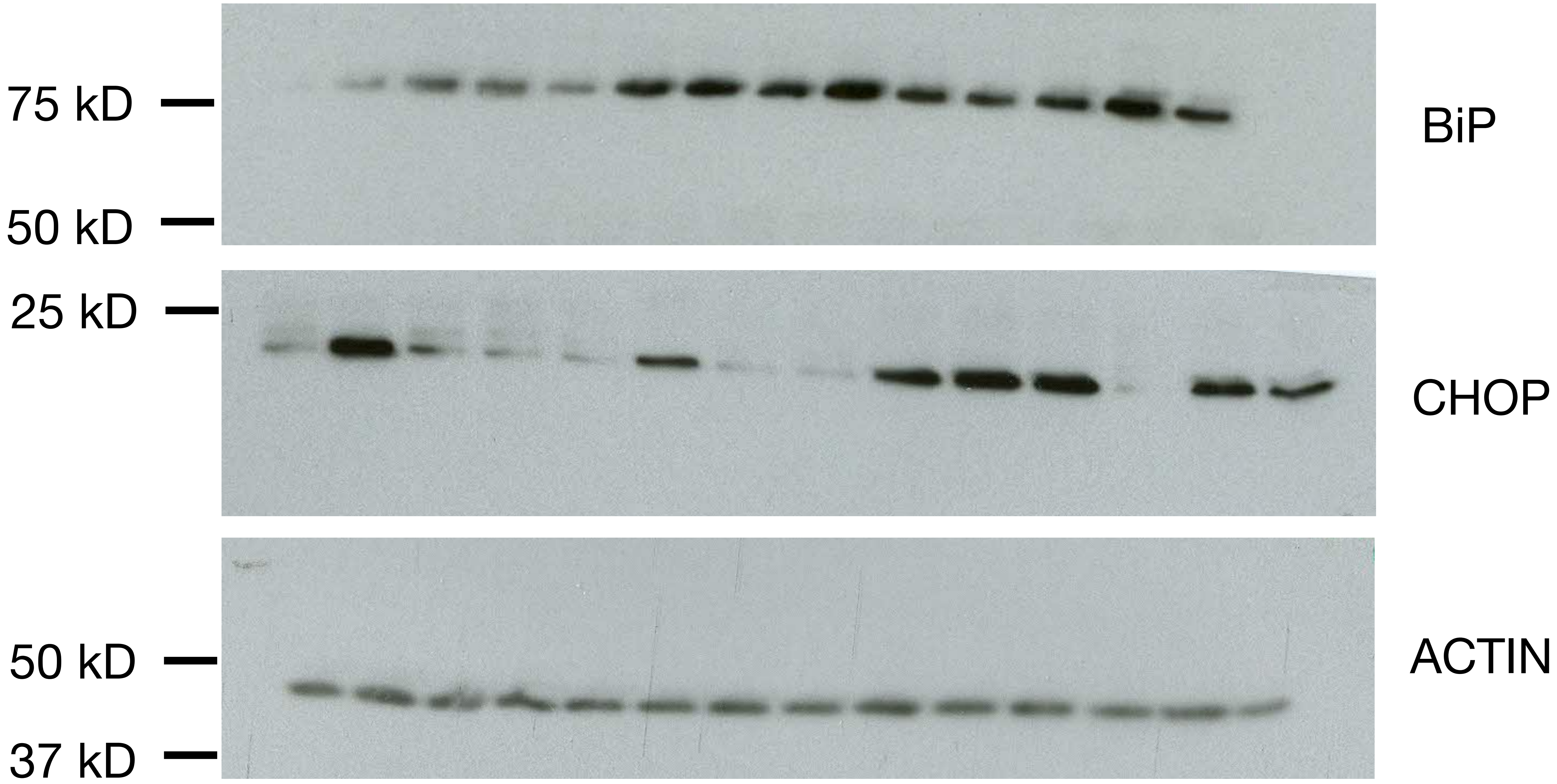

Figure 5G

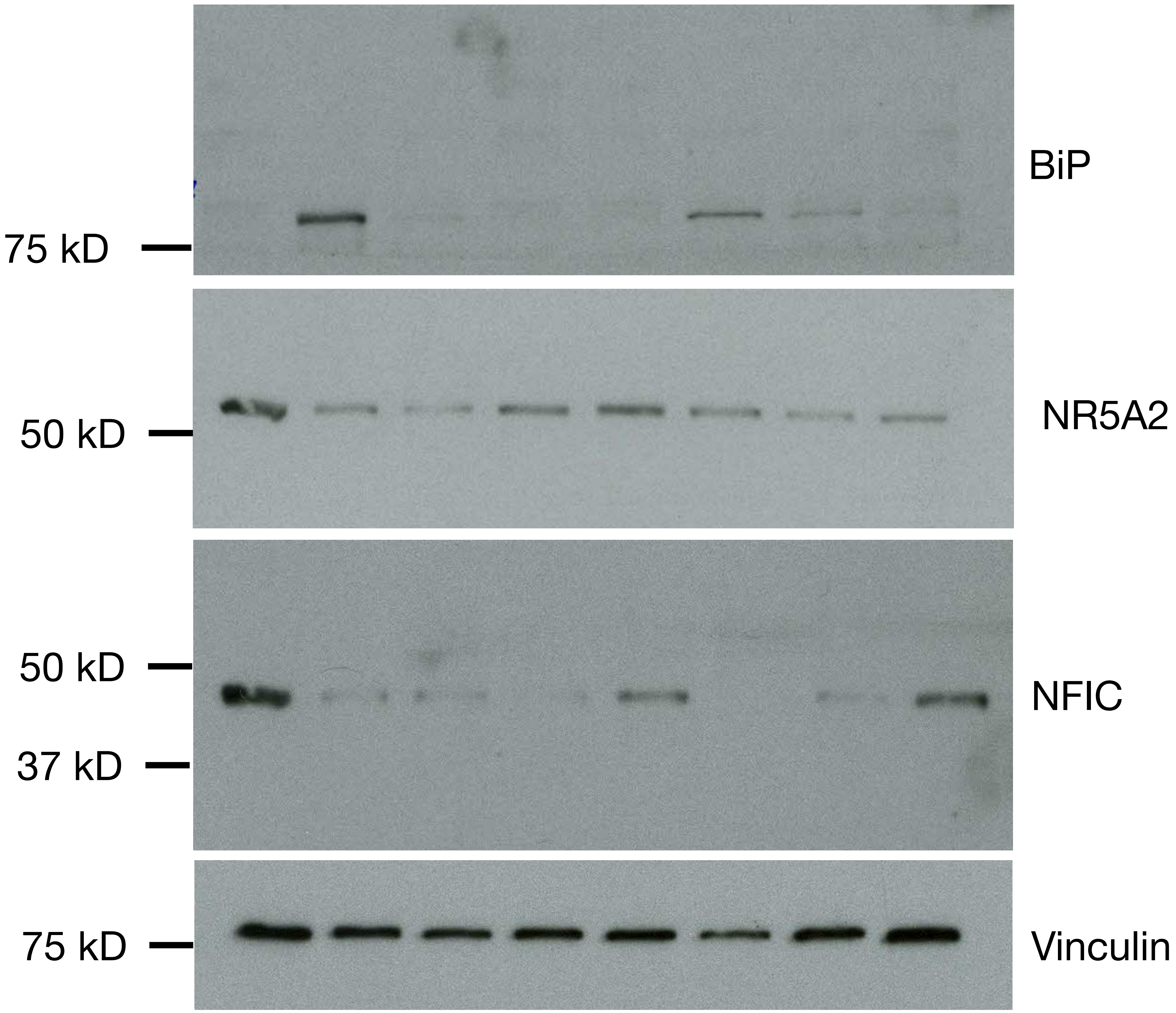

Figure 5H

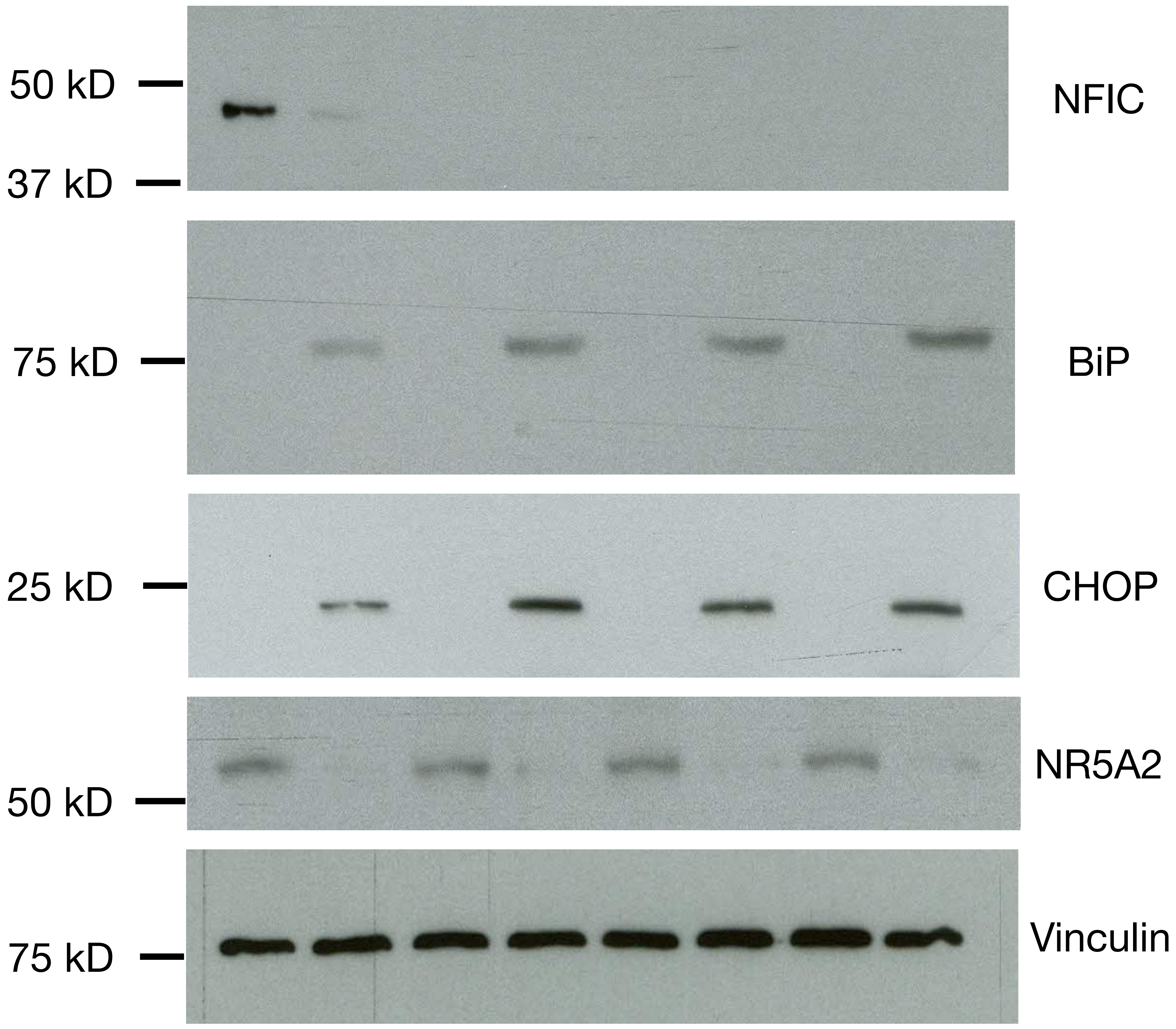

Figure 5I

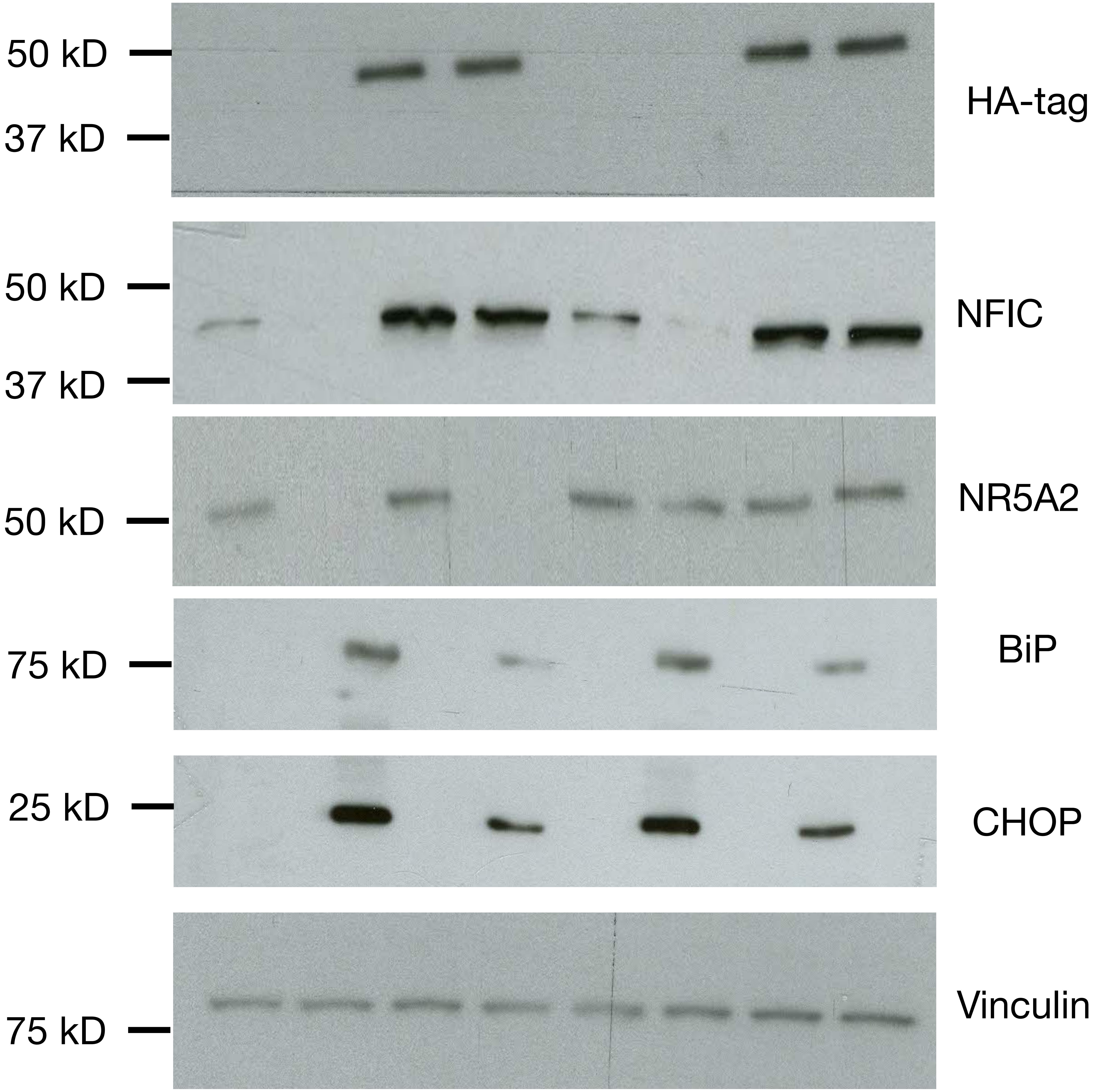
50 kD
37 kD
HA-tag
50 kD
37 kD
NFIC
50 kD
NR5A2
75 kD
BiP
25 kD
CHOP
75 kD
Vinculin
